# The Roles of tRNA-Derived Fragments in Cancer: Updates and Perspectives

**DOI:** 10.3390/ijms26125822

**Published:** 2025-06-17

**Authors:** Jiamian Geng, Zhaoyuan Sun, Hang Li

**Affiliations:** 1 Union Hospital, Tongji Medical College, Huazhong University of Science and Technology, Wuhan 430022, China; hustgjm@hust.edu.cn (J.G.); szy21@hust.edu.cn (Z.S.); 2 Department of Gastrointestinal Surgery, Union Hospital, Tongji Medical College, Huazhong University of Science and Technology, Wuhan 430022, China

**Keywords:** non-coding RNA, tRNA-derived fragments, biomarker, cancer

## Abstract

Non-coding RNAs (ncRNAs) and the significant roles they play in several diseases have been described and verified in numerous studies. Transfer RNA (tRNA)-derived fragments (tRFs) are a newly discovered class of small ncRNAs produced by mature or precursor tRNAs. In light of the development of RNA sequencing, evidence has shown that tRFs are widely involved in the generation and progression of diseases through a series of mechanisms, including RNA silencing, translational regulation, epigenetic regulation, reverse-transcriptional regulation, and cellular apoptosis. Several studies have determined that tRFs participate in several cancers in a number of ways. Furthermore, novel tRFs may hold significant potential as both diagnostic biomarkers and therapeutic targets in clinical applications. In this review, we discuss the biogenesis and classification of tRFs, illustrate their fundamental functions, and summarize the most recent tRF-related discoveries pertaining to cancer.

## 1. Introduction

Non-coding RNAs (ncRNAs)—including long non-coding RNAs (lncRNAs) and small non-coding RNAs (sncRNAs)—and their involvement in various biological processes have been the focus of many studies over the last few decades [1]. There is increasing evidence that sncRNAs play critical roles in multiple regulatory processes, including transcription, post-transcription, and translation. Their involvement in complex mechanisms is vital to the development and progression of cancers, and their potential value as biomarkers and/or therapeutic targets has received increased attention [2,3].

With the development of high-throughput sequencing technology and the advances in bioinformatics analysis, in addition to endogenous siRNAs (endosiRNAs), microRNAs (miRNAs), and PIWI-interacting RNAs (piRNAs) [4], researchers have uncovered the existence of a new type of sncRNA derived from tRNAs, called tRNA-derived fragments (tRFs), also termed tRNA-derived small RNAs (tsRNAs) in previous studies. tRFs, 14 to 32 nt long single-stranded RNAs, are generated from mature tRNAs or precursor tRNAs at different sites under the control of a set of highly conservative and precise site-specific cutting mechanisms [1,5,6]. Emerging evidence indicates that tRFs may participate in cell proliferation, the priming of viral reverse transcriptases, the regulation of gene expression, RNA processing, the modulation of the DNA damage response, tumor suppression, and neurodegeneration [7]. Moreover, tRFs have emerged as key contributors to the development and progression of multiple human pathologies, including cancer [8,9], inflammation [10], metabolic disorder [11], infection [12,13,14], and neurodegenerative disease [15,16]. Here, we will review the biogenesis and classification of tRFs and demonstrate some of their major biological functions, as well as bring together recent reports on the roles of tRFs in different types of cancer.

## 2. tRF Biogenesis and Classification

**Figure 1:**
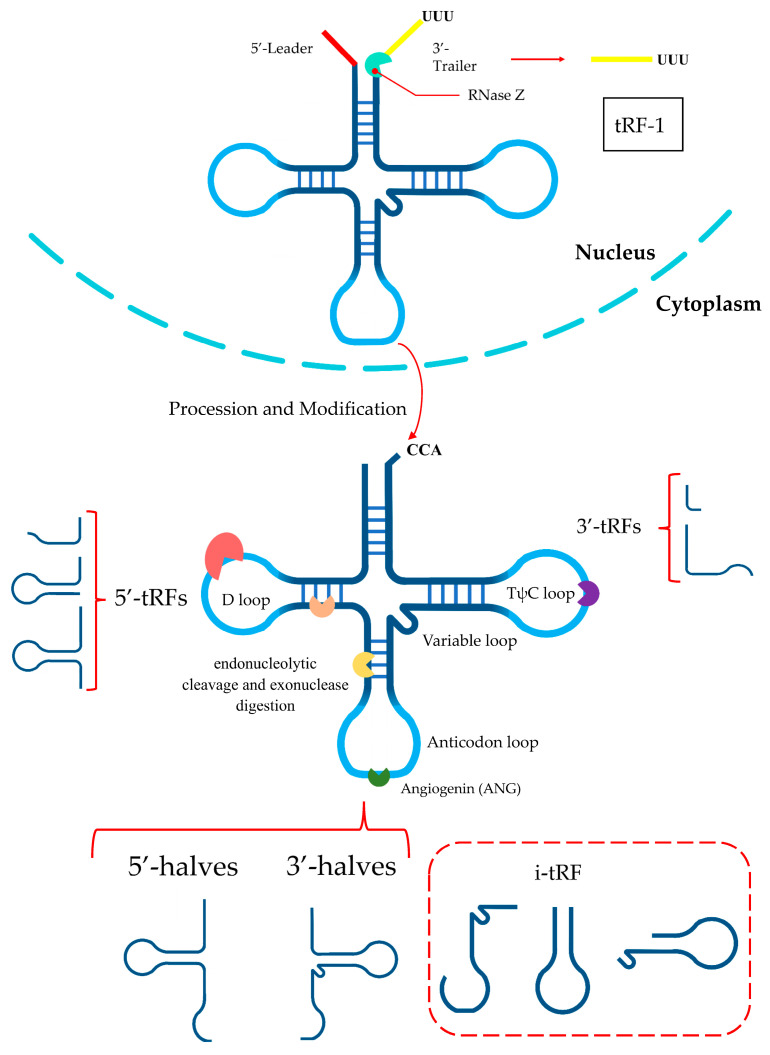
Different types of tRNA-derived RNA fragments. tRF-5s are produced by cleavage of the 5′ end in the D-arm, and tRF-3s are produced through cleavage of the 3′ end in the TψC arm. tRNA halves are generated through the cleavage of mature tRNAs at the anticodon arm by ANG. i-tRFs are a new type of tRF that originates from the internal zone of mature tRNAs. tRF-1s are produced by tRNA maturation.

tRNAs undergo comprehensive processing and a series of chemical modifications throughout their life cycle. In eukaryotes, pre-tRNAs undergo ribonuclease P and ribonuclease Z cleavage of the 5′ leader and 3′ tail sequences, respectively [17,18]. The addition of the CCA tail is catalyzed by the CCA-adding enzyme (tRNA nucleotidyltransferase) [19]. This is accompanied by further modifications, ultimately leading to the formation of mature tRNA. The structure of mature tRNA is highly conserved and characterized by a distinctive configuration comprising four arms (stem and loop)—the D-arm, anticodon arm, TψC arm, and acceptor arm—as well as the variable arm at the connection of the anticodon arm and the TΨC arm [20,21]. Mature tRNAs or pre-tRNAs can be cleaved at specific positions by corresponding ribonucleases, thereby generating a diverse array of tRFs [22,23,24]. tRNAs can generate five subtypes of tRFs, which are classified as follows: tRF-5 (5′-tRF), tRF-3 (3′-tRF), i-tRF, tRNA halves, and tRF-1. The former four types of tRFs are derived from mature tRNAs, and tRF-1s come from pre-tRNAs. The generation and classification of tRFs are shown in Figure 1.

tRF-5s are produced by cleavage of the 5′ end in the D-arm and tRF-3s are produced through cleavage of the 3′ end in the TψC arm [25,26]. tRF-5s can be classified into three specific subtypes according to their length: (i) type a (14–16 nt), (ii) type b (22–24 nt), and (iii) type c (28–30 nt). The cleavage sites for types a, b, and c are located in the D loop, D stem, and the 5′-half of the anticodon stem [5,27]. tRF-3s can be cleaved into two subcategories: tRF-3a and tRF-3b. The difference between the two is that members of the tRF-3b family are ~4 nt longer than those in the tRF-3a family; the approximate lengths of tRF-3a and tRF-3b are 18 and 22 nt, respectively [5,28]. Generally, tRF-5s and tRF-3s are generated in a Dicer-dependent fashion [14,28]; however, recent evidence has revealed that angiogenin (ANG) and other members of the ribonuclease A superfamily are actively involved in the biogenesis of these tRFs [28,29].

i-tRFs are a new type of tRF that originates from the internal zone of mature tRNAs. Different starting positions of cleavage define the different i-tRF subtypes. Based on the location of the 5′ terminus along mature tRNA, in one study, i-tRFs were divided into six subtypes [30]. There are other classification methodologies as well. The generation of i-tRFs may be associated with some specific conditions, such as hypoxia [31]. The biogenesis and generation of i-tRFs have not been demonstrated in their entirety and require further research.

tRNA halves, also defined as tRNA-derived stress-induced RNAs (tiRNAs), are generated through the cleavage of mature tRNAs at the anticodon arm by ANG. They can be divided into 5′-halves and 3′-halves [32]. 5′-halves have 30–35 nts ranging from the 5′ end to the anticodon loop, while 3′-halves have 40–50 nts from the anticodon loop to the 3′ end [6,26]. As a stress-inducible ribonuclease, ANG has a biological function that is strongly correlated with cellular status. The specific cleavage of tRNAs by ANG is typically induced under various stress conditions, including heat shock, ultraviolet irradiation, hypoxia, arsenite exposure, a lack of amino acids, and viral infection, leading to the generation of tRNA halves [26,33,34,35]. One study found that ANG is not the only RNase that produces tRNA halves, as other RNases may also be involved in this process [36]. In addition, tRNA halves are often produced disproportionately, leading to a much greater number of 5′-halves than 3′-halves [37].

Distinct from other tRFs, tRF-1s are produced by tRNA maturation, which originates from the 3′ trailer fragment of tRNA before the addition of the CCA tail trimmed by endonuclease Z (RNaseZ/ELAC2) [23,38]. At 16–48 nt in length, tRF-1s begin downstream of the 3′ end in pre-tRNA and end with a polyuridine sequence (UUUUU, UUCUU, AUCUU, or GUCUU) [23,39]. They are almost cytoplasmic, meaning they undergo translocation following their biogenesis in the nucleus [40].

## 3. Biological Functions of tRFs

**Figure 2:**
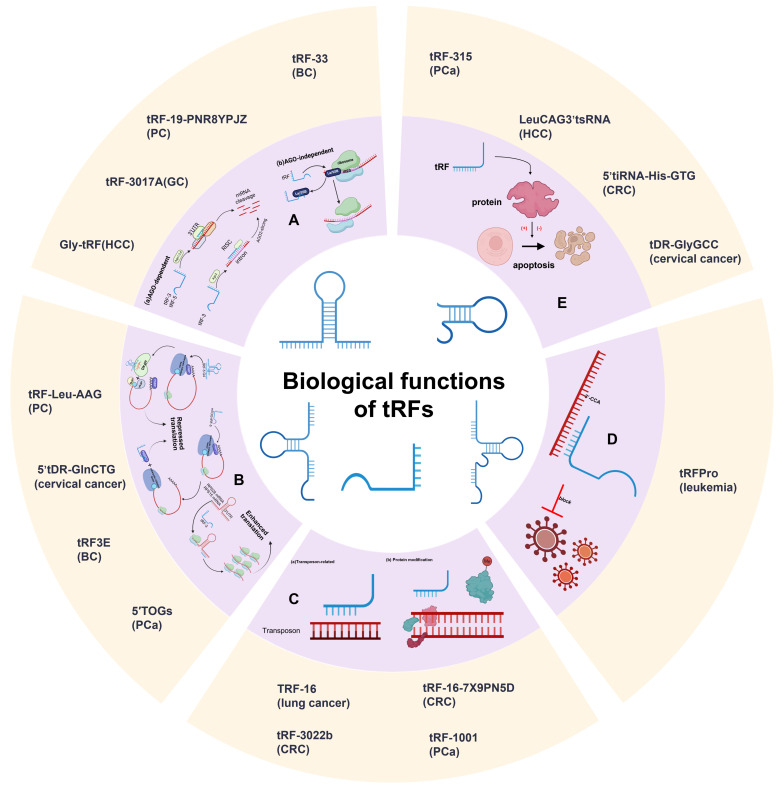
Functions of tRFs in diverse cancers. (**A**) RNA silencing; (**B**) translational regulation; (**C**) epigenetic regulation; (**D**) reverse-transcriptional regulation; and (**E**) cellular apoptosis.

The increasing depth of RNA sequencing has led to advances regarding the biogenesis and categorization of tRFs, which gives us an opportunity to explore their fundamental functions. Although the biological functions of tRFs are complicated and require additional investigation, based on our current knowledge, they can be divided into five categories: RNA silencing, translational regulation, epigenetic regulation, reverse-transcriptional regulation, and cellular apoptosis (Figure 2).

### 3.1. RNA Silencing

Recent studies have cast more light on tRFs’ important roles in RNA interference-mediated silencing, with several indicating that some tRFs show distinct affinities with various Argonaute (AGO) subtypes. A meta-analysis of PAR-CLIP data revealed that tRF-5s and tRF-3s are associated with AGO1, 3, and 4, rather than AGO2, and the analysis of positional T to C mutational frequency indicated that the pattern of the association between tRFs and AGOs is similar to that of miRNAs [5]. For example, Green’s group illustrated that tRF-3003a, produced by the cleavage of tRNA-CysGCA, confers gene silencing of Janus Kinase 3 (JAK3) via AGO/RISC formation in osteoarthritis chondrocytes [41]. In contrast to microRNAs, tRFs exhibit a distinct subcellular localization, predominantly within the cytoplasmic compartment, and demonstrate selective binding to AGOs, while notably lacking association with MOV10. Moreover, tRF-3s exhibit a canonical microRNA-/siRNA-like trans-silencing capacity, while tRF-1 does not exhibit apparent trans-silencing activity [38]. A further study shows that Dicer-dependent tRFs promote gene silencing through a mechanism distinct from PTGS and TGS, which can lead to the downregulation of target genes by targeting introns via nascent RNA silencing (NRS) in nuclei [42]. In addition to the AGO-dependent mechanism for gene silencing by tRFs, AGO-independent mechanisms, including competitive binding of target proteins with messenger RNAs (mRNAs), have also been found. Hee et al. demonstrated that RF-U3-1 inhibits HCV IRES-mediated translation by sequestering the limiting amounts of La/SSB available in the cytoplasm via La/SSB knockout Huh7 cell lines [43]. Hence, tRFs might be involved in multiple pathways regulating RNA silencing; discovering these pathways is urgently needed.

### 3.2. Translational Regulation

tRFs can modulate translational processes by regulating translation initiation, elongation, and ribosome biogenesis, thereby exerting an influence on overall protein synthesis. The involvement of tRFs in translation regulation was initially discovered in stress-related conditions [32]. It has been identified that transfection of natural 5′-halves, but not 3′-halves, results in the suppression of global translation in *U2OS* cells [32]. As previously mentioned, cellular stress leads to the production of tRNA halves by ANG. These specific 5′-tiRNAs, such as 5′-tiRNAAla and 5′-tiRNACys, can potentially displace the mRNA cap-binding protein, eukaryotic translation initiation factor 4E (eIF4E) [44]. Further research has demonstrated that this restraint is achieved by the aggregation of 5′-tiRNAs into tetrameric G-quadruplexes (G4) mediated through the 5′ terminal oligoguanine (5′ TOG) motif [45,46]. G4-tiRNAs, which are necessary for tRFs in the regulation of mRNA translation, can directly bind to the HEAT1 domain of eukaryotic translation initiation factor 4G (eIF4G) [45,47]. This displacement can induce the phospho-eIF2α independent assembly of stress granules (SGs), leading to the failure of the translation initiation scanning process and consequently suppressing global translation [48].

Besides tRNA halves, tRF-5s can also affect translation. One study demonstrated that, in embryonic stem cells, the Ψ modification at U8 mediated by pseudouridine synthase 7 (PUS7) can motivate TOG-containing tRF-5s to bind to polyadenylate-binding protein 1 (PABPC1), a protein in charge of eIF4G/A interaction and initiation of cap-dependent translation. The mTOG-PABPC1 complex can intercept PABPC1 recruitment to eIF4F, thus blocking translation [49]. Additionally, a general conserved “GG” dinucleotide in tRF-5s has also been shown to participate in the process of protein translation [50].

An important part of the production of tsRNAs is the 5-methylcytosine (m5C) modification. NOP2/Sun domain 2 (NSUN2) is a specific cytosine-5 RNA methyltransferase of proteins that methylates the cytidine residues of most tRNAs. In the absence of NSUN2, the loss of cytosine-5 RNA methylation increases the cleavage of tRNA, resulting in an accumulation of 5′-tRFs. These abnormal processes eventually reduce protein translation rates [37].

In addition, tRFs can also promote protein generation through strengthening translation-induced ribosome biogenesis. Binding at least two ribosomal protein (RPS28 and RPS15) mRNAs, a kind of 3′-tRF from tRNALeu (CAG) can enhance translation [51].

### 3.3. Epigenetic Regulation

The regulation of genetic expression is mediated through an integrated interplay between genomic DNA sequences and epigenetic regulatory mechanisms. Epigenetics mainly regulates gene expression through DNA methylation, histone modification, nucleosome remodeling, and ncRNA regulation [52]. Previous studies have highlighted evidence suggesting that epigenetic misregulation can culminate in human diseases, such as cancer [53]. Here, we will summarize the potential epigenetic mechanisms of tRFs, dividing them into two basic categories: PIWI-independent and PIWI-dependent epigenetic regulation.

Transposons are genetic sequences that can translocate their sites within a genome [54]. Together with their repetitive sequences, they contribute to the formation and function of chromosomes and induce the epigenetic regulation of specific genes. Because of the high mobility and mutation of transposons, eukaryotic cells have evolved retiform epigenetic mechanisms in order to preserve genetic integrity [55]. In agreement with this, substantial evidence has shown that various cancers are significantly correlated with the transcriptional activity of transposons. Recently, Andrea et al. discovered that tRF-3s (18 and 22 nt in length) containing the 3′ terminal CCA sequence of mature tRNAs could inhibit long terminal repeat (LTR) retrotransposons (also known as endogenous retroviruses (ERVs)) through different mechanisms involving sequence complementarity with the primer binding site (PBS) sequence [56]. Furthermore, Sharma et al. found that 5′-tRF-Gly-GCC (tRF-GG) affects chromatin accessibility in murine endogenous retrovirus-L (MERVL) elements, as well as throughout heterochromatin in embryonic stem (ES) cells and preimplantation embryos [57]. Their discovery suggests that the underlying mechanisms of interactions between tRFs and chromatin remain to be elucidated, thereby offering novel insights for future investigations.

Next, we will briefly introduce the PIWI-dependent epigenetic regulation of tRFs. Couvillion et al. revealed a potential connection between tRF-3s, 18–22 nt long tsRNAs with the predominant 5′ end in the TΨC loop and the 3′ end at the mature tRNA 3′ terminus, and the *Tetrahymena thermophila* Ago/PIWI protein Twi12, highlighting their essential roles in epigenetic regulation [58]. They discovered that tRF-3 is implicated in the nuclear translocation of Twi12, while Twi12 plays an essential role in ribosomal RNA processing by assembling with Xrn2 and Tan1 proteins [58]. Another study showed that IL-4 potently decreases the biogenesis of PIWI-interacting RNAs (piRNAs), specifically tRNA-Glu-derived piRNAs [td-piR(Glu)]. Further, Xue Zhang et al. revealed that the td-piR(Glu)/PIWIL4 complex recruits SETDB1, SUV39H1, and heterochromatin protein 1b to the CD1A promoter region and facilitates heterochromatin histone H3 Lys9 (H3K9) methylation, leading to a significant inhibition of CD1A transcription [59]. These results suggest that td-piR(Glu) participates in chromatin remodeling in immune cells.

### 3.4. Reverse-Transcriptional Regulation

Some of the roles of tRFs in reverse transcription have also been demonstrated by studies in the field of gene expression and oncology [60]. A study conducted in mice has shown that 3′-tRFs are crucial regulators of the retrotransposons in the cell [61]. Transposable elements (TEs) actuate transcription, and in mice, the most active transposons are ERVs. There is a large number of 3′-tRFs attacking ERVs in mouse stem cell lines. The 18 nt 3′-tRFs specifically restrain ERV activity by competing with tRNAs for the highly conserved PBS of ERVs, resulting in the blockade of reverse transcription. Notably, the 22 nt 3′-tRFs can also bind to the PBS sequence, which affects transposon expression by post-transcriptionally silencing ERV mRNA [61]. Both of these 3′-tRFs inhibit ERV activity through different mechanisms and both depend on base complementarity with the PBS sequence.

In viral—especially retrovirus—infection, tRFs can affect reverse transcription. Human T-cell lymphotropic virus Type I (HTLV-1) is the first human retrovirus ever discovered [62]. In infected CD4 cells, tRF-3s are considerably more abundant than other tRFs [63]. Among tRF-3s, tRF-3019 corresponds to the 3′ end of tRNA-Pro, which is the tRNA considered to be the primer for HTLV-1 reverse transcriptase [64]. The portion of tRNA-Pro corresponding to tRF-3019 is complementary to the HTLV-1 PBS, which may indicate that this tRF would be fully sufficient as a primer for reverse transcription. It could thus induce HTLV-1 to initiate reverse transcription and accumulate viral replication [60,63].

### 3.5. Cellular Apoptosis

Recent studies have indicated the potential role of tRFs in regulating cell apoptosis. While previous reports demonstrated that apoptosome formation in vitro was inhibited by the addition of in vitro-synthesized full-length tRNAs [65,66,67], subsequent research revealed that ANG-induced accumulation of tRNA halves is associated with increased survival in hyperosmotically stressed mouse embryonic fibroblasts via cell apoptosis inhibition. The underlying mechanism is that **cytochrome c** (Cyt c), released from the mitochondria during hyperosmotic stress, forms RNPs with tRNAs, which disturb the formation of the apoptosome by interfering with the interaction of apoptotic protease activating factor 1 (APAF1) and Cyt c. Saikia et al. revealed a novel signaling pathway that interferes with Cyt c-mediated caspase activation, a novel function of tiRNAs as inhibitors of apoptosome formation and function, and a novel antiapoptotic mechanism involving ANG-mediated tRNA cleavage. However, how Cyt c recognizes specific tRNA targets needs further research [68].

Another study utilizing whole-transcriptome sequencing technology discovered a novel tRF, tRF-3022b, which shows an increasing trend in colorectal cancer (CRC) tissues compared to adjacent normal tissues. tRF-3022b plays a vital role in apoptosis inhibition and CRC progression through the regulatory pathway by binding to galectin 1 (LGALS1) and macrophage migration inhibitory factor (MIF) in CRC cells [69]. However, tRFs do not consistently act as oncogenic drivers. Ling Pan et al. identified an inflammatory cytokine-regulated tRFs, tRF-21-VBY9PYKHD (tRF-21), which suppresses the progression of pancreatic ductal adenocarcinoma (PDAC) by enhancing apoptosis. Further research revealed that tRF-21 knockdown promotes the phosphorylation of heterogeneous nuclear ribonucleoprotein L (hnRNP L) and the formation of hnRNP L and dead-box helicase 17 (DDX17) complexes. These complexes play crucial roles in splicing Caspase 9 into Caspase 9b (with antiapoptotic specificity) and mH2A1.2 (with pro-invasive specificity), while tRF-21 upregulation exerts the opposite effect [70].

## 4. tRFs in Cancer

**Figure 3:**
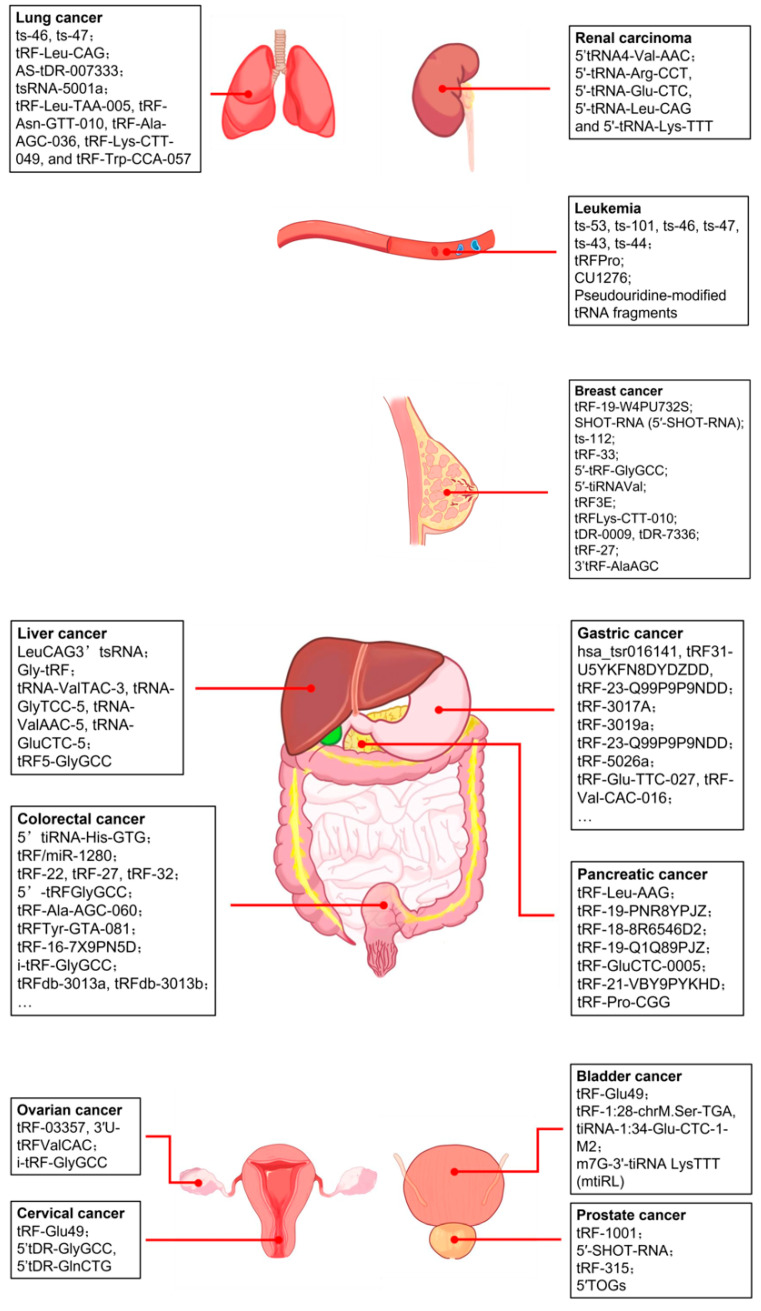
Functions of tRFs in different kinds of cancer. tRFs are associated with many types of cancer, including breast cancer, prostate cancer, pancreatic cancer, liver cancer, gastric cancer, colorectal cancer, leukemia, lung cancer, and other cancers. These tRFs play distinct roles in different types of cancer through various mechanisms.

Deep sequencing and microarrays have clarified the functions of tRFs in specific diseases and promoted the development of several tRF-related databases [51,71]. Further research has substantiated the involvement of tRFs in a multitude of biological mechanisms that are intricately linked to oncogenesis and the advancement of malignancies (Figure 3). Given that the interactions between different cancer cells and their respective internal tRFs are highly heterogeneous, we will summarize the latest studies of the functions and potential mechanisms of tRFs in diverse cancers, as well as their deployment in clinical practice. Related contents have been systematically summarized in Table S1.

### 4.1. Breast Cancer

Breast cancer (BC) is the most common cancer referenced in tRF studies, and the role of tRFs in BC has received increasing attention over the past few years. tRFs are promising candidate diagnostic and prognostic biomarkers for BC, as well as possible therapeutic targets. Some studies have found that specific tsRNAs in plasma could play crucial roles as diagnostic and prognostic biomarkers in BC. Circulating tRF-ArgCCT-017, tRF-Gly-CCC-001, and tiRNA-Phe-GAA-003 are upregulated in plasma samples of BC patients, whereas 5′-tRF-His-GTG is downregulated, which indicates their potential as biomarkers [72,73]. By using a custom tsRNA microarray chip, one study found signatures of 34 tsRNAs able to distinguish normal cells from BC cells; the expression of these tRFs also varied in two cell lines at different stages of BC, suggesting that tRFs may play a regulatory role during tumor development [74].

Different kinds of tRFs have various functions in BC tumorigenesis and progression. A few tRFs have been found to act as enhancing factors in BC. Through the regulation of ribosomal protein, tRF-19-W4PU732S from mature tRNA-Ser-AGA can affect BC by inhibiting ribosomal protein-L27A (RPL27A), eventually promoting cell proliferation, malignant activity, invasion, the EMT, cancer stem cell (CSC) phenotypes, and the suppression of apoptosis [75]. Sex hormone-dependent tRNA-derived RNAs (SHOT-RNAs) are specifically expressed in ER-positive BC cell lines; similarly, 5′-SHOT-RNA can also accelerate cell proliferation in BC cells [76]. Farina et al. demonstrated ts-112 to be an oncogenic tRF selectively inhibited by the tumor suppressor runt-related transcription factor 1 (RUNX1) to prevent overactive expression in breast epithelial cells [77]. Recently, it was discovered that tRF-33 from mature tRNA-LysTTT was significantly upregulated in human epidermal receptor 2 (HER2)-negative BC cells and tissue. By directly interacting with the 3′-UTR of insulin-like growth factor 1 (IGF1) mRNA, tRF-33 was identified to lead to the downregulation of IGF1 mRNA expression and protein synthesis, eventually disrupting mitochondrial homeostasis and contributing to the progression of BC pathogenesis [78]. In another study, tRFs were verified to affect BC through an epigenetic mechanism. By increasing fat mass and obesity-associated protein (FTO) demethylase activity and reducing the m6A levels of eukaryotic translation initiation factor 4 gamma 1 (eIF4G1), overexpressed 5′-tRF-GlyGCC could decrease autophagy and promote tumorigenesis and metastasis in BC cells [79].

Some tRFs that act as tumor suppressors have also been identified. Under hypoxic stress, a new class of tRFs is produced from tRNAGlu, tRNAAsp, tRNAGly, and tRNATyr [31]. This class of tRFs has a motif corresponding to Y-box binding protein 1 (YBX1), which is implicated in cancer progression and has been shown to promote cancer metastasis [80,81]. These tRFs can inhibit the development of BC metastases by binding YBX1 and displacing multiple oncogenic transcripts such as EIF4EBP1 and AKT1. One study showed that 5′-tiRNAVal, as a new tumor suppressor, can directly target the 3′-UTR sequence in frizzled class receptor 3 (FZD3) and inhibit the FZD3-mediated Wnt/β-catenin signaling pathway in BC cells [82]. Furthermore, its downregulation in serum was positively related to BC pathological stage and lymph node metastasis. Coming from the 3′ end of mature tRNAGluTTC, tRF3E is specifically expressed in healthy mammary glands but not in BC. This tRF specifically interacts with nucleolin (NCL) and causes the release of p53 mRNA, promoting its translation by competing for NCL and eventually resulting in an inhibition of BC cell proliferation through this mechanism [83].

Triple-negative breast cancer (TNBC) is the most aggressive and malignant type of BC, characterized by restricted treatment options, chemoresistance, and poor prognosis. An analysis of the tRF expression profiles in CSCs isolated from TNBC and non-TNBC cell lines found that tDR-000620 can independently act as a novel candidate biomarker for the early detection of recurrence in TNBC patients, with a continuously lower expression level in TNBC CSCs and serum samples [84]. tRFLys-CTT-010 is significantly increased in TNBC and promotes TNBC proliferation and migration. tRFLys-CTT-010 interacts with the glucose-6-phosphatase catalytic subunit (G6PC) to regulate lactate production and glycogen consumption, leading to cell survival and proliferation [85]. This indicates that adjusting glucose metabolism and the tRFLys-CTT-010/G6PC axis may provide innovative therapeutic targets for TNBC treatment.

In the treatment of BC, modulating tRFs may represent a promising approach to suppressing tumor growth and progression. Several studies have demonstrated that tRFs could affect the drug resistance of BC cells. Under hypoxia, tDR-0009 and tDR-7336 were found to be involved in maintaining the stem cell population and the cellular response to IL-6, thereby facilitating doxorubicin resistance in TNBC [86]. Moreover, in HER2-positive BC patients, trastuzumab, a specific monoclonal antibody, has prolonged the survival of these patients immensely [87]. Overexpression of tRF-27 competitively binds to Ras GTPase-activating protein-binding proteins 1 and 2 (G3BPs) and activates the mechanistic target of rapamycin complex 1 (MTORC1) to enhance cell proliferation and induce the resistance of HER2-positive BC against trastuzumab [88]. Meanwhile, the potential of tRFs to alleviate chemoradiotherapy resistance is still noteworthy. It has been discovered that silencing 3′tRF-AlaAGC enhanced the sensitivity of BC cells to Adriamycin through the NF-κb signaling pathway [89].

### 4.2. Prostate Cancer

Prostate cancer (PCa) is the third leading cause of cancer-related death among men worldwide [90]. Previously, a study reported the discovery and differential expression of tRFs in clinical samples of PCa [91]. Michael et al. used RNA-sequencing to analyze the expression of tRFs in fresh-frozen patient samples derived from normal adjacent prostate and different stages of PCa and found that tRF-5s comprise the most abundant class of tRFs in general and represent a major class among upregulated tRFs. On the contrary, the tRF-3 type is predominant among downregulated tRFs in PCa [92]. An analysis of tRF profiles from the Prostate Adenocarcinoma (PRAD) dataset in The Cancer Genome Atlas (TCGA) found that tRFs have extensive correlations with mRNAs, which are disrupted in PRAD [93]. The studies cited above indicate that tRFs might play an important role in the pathogenesis of cancer.

SHOT-RNAs are specifically and abundantly expressed in androgen receptor (AR)-positive PCa cell lines, which are produced from amino-acylated mature tRNAs by ANG-mediated anticodon cleavage, promoted by sex hormones and their receptors. In one study, the researchers transfected siRNA targeting 5′-SHOT-RNA^LysCUU^ and found a severe impairment of cell proliferation compared with the control siRNA-transfected cells, indicating the enhancer roles of SHOT-RNAs in cell proliferation [76]. tRF-1001, a specific tRF-1 generated by the PCa susceptibility gene tRNA 39-endonuclease ELAC2, is also involved in cell proliferation in PCa [23].

As for prostate cancer treatment, platinum-based therapies serve as a valuable adjunct to anti-androgen therapies, particularly in the management of small-cell PCa (SCPC) [94]. In PCa, cisplatin inhibits cell growth and induces apoptosis in both a P53-dependent and -independent manner [95]. However, the potential mechanism of platinum resistance requires further investigation. tRF-315, derived from tRNALys, prevents cisplatin-induced apoptosis and alleviates cisplatin-induced mitochondrial dysfunction in PCa cells (*LNCaP* and *DU145*) by inhibiting the cisplatin-induced upregulation of the p53-GADD45A axis. Thus, tRF-315 diminishes the therapeutic effect of cisplatin in PCa [96].

Moreover, growing evidence has highlighted that methyltransferase-like protein-1 (METTL1) depletion causes the loss of m7G tRNA methylation and promotes the biogenesis of 5′ terminal oligoguanine-containing tRNA fragments (5′ TOGs), which regulate the activation of the interferon signaling pathway in cancer cells and transform the immunosuppressive prostate tumor microenvironment (TME) into a tumoricidal endotype, providing novel insights into PCa therapeutic strategies, particularly for patients with castration-resistant PCa [97].

### 4.3. Pancreatic Cancer

Extensive research has revealed the pivotal role and clinical potential of tRFs in pancreatic cancer (PC). The analysis of cancerous and adjacent normal tissues from PC patients using high-throughput second-generation sequencing techniques revealed that the expression of tRF-Leu-AAG is observably upregulated in PC tissues. Further research has shown that tRF-Leu-AAG decreases the level of up-frameshift protein 1 (UPF1), one of the core proteins of nonsense-mediated mRNA decay (NMD), which is positively correlated with cell proliferation, migration, and invasion [98]. Similarly, tRF-19-PNR8YPJZ promotes PC migration and invasion by acting through the axis inhibition protein 2 (AXIN2) axis; the overexpression of this receptor activated the Wnt/β-catenin pathway by decreasing AXIN2 expression [99]. Moreover, tRF-18-8R6546D2, a novel oncogenic factor, promotes PC malignancy partly by directly silencing achaete-scute homolog 2 (ASCL2) and further regulating its downstream genes such as *MYC* and *CASP3* [100]. On the contrary, tRF-19-Q1Q89PJZ suppresses the malignant activity of PC cells by regulating hexokinase 1 (HK1)-mediated glycolysis. In a subcutaneous xenograft model, tumor tissues with tRF-19-Q1Q89PJZ knockout exhibited a significantly reduced growth rate, tumor mass, and tumor marker levels compared to non-knockout controls. The results of a pulmonary metastasis model indicated that the knockdown group exhibited higher metastatic ability and shorter survival times in survival analyses [101].

As for pancreatic ductal adenocarcinoma (PDAC), the upregulation of tRF-GluCTC-0005 enhances the mRNA stability of WD repeat domain 1 (WDR1), thereby promoting cancer cell proliferation, migration, and invasion [102]. tRF-GluCTC-0005 also plays a crucial role in PDAC liver metastasis by remodeling the TME. Wei Chen et al. used mouse models established via the tail vein injection of 2 × 10^6^ Panc02 cells and discovered that treatment with the PDAC exosome for four weeks led to a significantly enhanced liver metastatic propensity in the tRF-GluCTC group compared to the control group. The underlying mechanism is that the high level of tRF-GluCTC-0005 in peripheral blood serum exosomes binds to the 3′ untranslated region of the WDRl mRNA in hepatic stellate cells (HSCs), upregulating the translation of WDRl. Then, the N-terminal of WDR1 binds to YAP, activating HSCs, promoting hepatic fibrosis and myeloid-derived suppressor cells (MDSCs) infiltration, and creating a favorable microenvironment for PC liver metastasis [103]. On the other hand, tRF-21-VBY9PYKHD (tRF-21), a tumor suppressor in PDAC, promotes cancer cell proliferation, migration, and invasion when downregulated. Its downregulation provokes hnRNP L-DDX17 activity, leading to the formation of Caspase 9b and mH2A1.2, thereby promoting PDAC cell malignant phenotypes [70]. Studies also highlight the role of tRFs as potential biomarkers for PDAC prognosis and therapy. Jun Li et al. revealed that a significant downregulation of tRF-Pro-CGG is associated with an advanced TNM stage (*p* = 0.000) and N stage (*p* = 0.000) in patients. More importantly, low tRF-Pro-CGG expression predicts poor survival in PDAC patients (*p* = 0.003) [104].

### 4.4. Liver Cancer

At present, research on the role of tRFs in liver cancer, especially their molecular mechanisms, is still lacking. A specific tRF-3 derived from Leu-CAG tRNA (LeuCAG3′tsRNA) was reported in 2017. Its inhibition induced apoptosis in *HeLa* and *HCT-116* cells in vitro and in a patient-derived orthotopic hepatocellular carcinoma (HCC) model in mice. The underlying molecular mechanism is that LeuCAG3′tsRNA interacts with RPS28 and RPS15, two ribosomal protein mRNAs, to enhance their translation and increase tumor cell viability [51]. Recent studies reported that glycine tRNA-derived fragment (Gly-tRF), generated from the 5′-arm of glycine tRNA, was highly expressed in HCC cell lines and tumor tissues. Gly-tRF increased the liver CSC (LCSC) subpopulation proportion and LCSC-like cell properties, in addition to promoting the epithelial–mesenchymal transition (EMT). Mechanistically, Gly-tRF decreased the level of Nedd4 family-interacting protein 2 (NDFIP2) mRNA by binding to the NDFIP2 mRNA 3′ UTR and activating the AKT signaling pathway [105]. Carcinoembryonic antigen-related cell adhesion molecule 1 (CEACAM1) is another direct target of Gly-tRF. 5′tRF-Gly promotes HCC cell migration and proliferation by inhibiting the translation of CEACAM1 by directly targeting the 3′-UTR [106]. Conversely, updated evidence suggests that specific tRFs may exert protective effects in the pathogenesis of HCC. HCETSR—a novel tRF derived from tRNA-Glu/TTC—inhibits HCC malignancy by regulating the Spectrin, beta, non-erythrocytic 1 (SPBTN1)–catenin complex axis. Mechanistically, HCETSR disrupts the interaction between SPTBN1 and the catenin complex, as well as facilitating the transfer of the catenin complex from the cell membrane to the nucleus to inhibit oncogene LEF1 transcription [107]. Gly-tRF, along with HCETSR, could provide new targets for the prognostic evaluation and treatment of HCC.

Accumulating evidence supports the conclusion that tRFs possess promising clinical significance in HCC, demonstrating potential utility across diagnosis, prognosis monitoring, and therapeutic management. Yi Zuo et al. built a five-tsRNA-based random forest (RF) diagnostic model and a seven-tsRNA-based risk score signature for liver cancer prognosis using the LASSO model. The diagnosis model had an area under the receiver operating characteristic curve (AUROC) of 88% and an area under the precision–recall curve (AUPR) of 87%, whereas the prognostic model predicted the overall survival (OS) of liver cancer patients (Hazard Ratio 2.02, 95% CI 1.36–3.00, *p* < 0.001) independent of standard clinicopathological prognostic factors. Diagnostic and prognostic modeling based on tsRNA characterization can thus be reliably utilized in the diagnosis of liver cancer and in the classification of high- and low-risk patients [108]. Lei Zhu et al. discovered that patients with liver cancer exhibited significantly higher levels of four tsRNAs (tRNA-ValTAC-3, tRNA-GlyTCC-5, tRNA-ValAAC-5, and tRNA-GluCTC-5) in the plasma exosome [109]. This finding demonstrates that plasma exosomal tsRNAs could serve as novel diagnostic biomarkers for HCC.

HCC recurrence postresection represents a thorny problem in clinical practice [110]. A growing number of studies have revealed that radiotherapy can not only directly eliminate tumor cells but also enhance NK cell cytotoxicity by upregulating the NK group 2D ligand on tumor cells [111,112]. However, radiotherapy also suppresses the antitumor immunity of NK cells, which limits its therapeutic efficacy [113,114]. Yihang Gong et al. revealed that the glycocholic acid (GCA)/tRNA-derived fragment 5 (tRF5)-GlyGCC signaling axis is activated in a mouse HCC model after radiotherapy. tRF5-GlyGCC epigenetically upregulates Runx2 by interacting with histone 3 lysine 27 demethylase (KDM6B) and then transcriptionally activates integrin beta-like 1 (ITGBL1) and S100A9 expression in HCC cells, which further reduces NK cell cytotoxicity directly and attracts MDSCs to indirectly inhibit NK cell function [115]. These findings highlight the possibility of successfully combining radiotherapy with tRF5-GlyGCC inhibitor in order to potentiate NK cell antitumor immunity and prevent HCC recurrence postresection.

### 4.5. Gastric Cancer

Increasing numbers of tRFs involved in gastric cancer (GC) have been revealed. In GC tissues and serum, several studies have demonstrated that the overexpression of hsa_tsr016141, tRF31-U5YKFN8DYDZDD, and tRF-23-Q99P9P9NDD is closely related to tumor grade, lymph node metastasis, and invasion, respectively [116,117,118]. Derived from tRNA-Val-TAC, tRF-3017A regulated the invasion and migration of GC cells by forming an RNA-induced silencing complex (RISC) with AGO2 to target the silencing of the tumor suppressor gene *NELL2* [9]. Besides targeting the gene, another study showed that tRF-3019a, a kind of 3′-tRF, binds with the mRNA 3′UTR of the tumor suppressor gene F-box protein 47 (FBXO47), leading to GC cell proliferation, migration, and invasion [119]. Concurrently, certain upregulated tRFs can modulate metabolic processes within tumor cells. The overexpressed tRF-23-Q99P9P9NDD could bind to the 3′UTR site of acyl-coenzyme A dehydrogenase short/branched chain (ACADSB), thereby affecting GC lipid metabolism and ferroptosis and promote tumor progression [120].

Additionally, a significant number of downregulated tRFs have been identified in GC. Some special tRFs, including tRF-33-P4R8YP9LON4VDP and tRF-193L7L73JD, disrupt the cell cycle of GC cells at the G0/G1 phases, thereby suppressing their growth and proliferation [121,122]. Certain tRFs exert impacts on GC through their regulatory effects on signal transduction pathways. tRF-5026a inhibited the occurrence and development of GC through the PI3K/AKT signaling pathway; however, the expression of tRF-5026a observed in GC tissues and cells was low [123]. tRF-5026a was also found to potentially change cell cycle progression in GC. The MAPK signaling pathway is involved in the regulatory network orchestrated by GC-associated tRFs. tRF-Glu-TTC-027 can regulate the MAPK pathway, probably by targeting the 3′ UTR sites in transforming growth factor beta 2 (TGF-β2) [124]. Similarly, tRF-Val-CAC-016 directly targets the Calcium Voltage-Gated Channel Subunit Alpha1 D (CACNA1d) to regulate the MAPK signaling pathway in GC progression [125]. Meanwhile, tRF-Tyr binds directly to the heterogeneous nuclear ribonucleoprotein D (hnRNPD) protein and competitively displaces the binding of hnRNPD to the c-Myc 3′UTR, regulating the c-Myc/Bcl2/Bax pathway and ultimately inhibiting the progression of GC [126].

Utilizing these tRFs in the clinical detection of GC is currently under exploration. A study has put forward that a kind of serum 3′-tRF can act as a potential diagnostic biomarker in this regard, due to its high diagnostic efficacy with other tumor markers [127]. tRF-Val, a kind of 3′tRF, directly binds to eukaryotic translation elongation factor 1 alpha 1 (EEF1A1), mediates its transport into the nucleus, and promotes its interaction with murine double minute 2 (MDM2), a specific p53 E3 ubiquitin ligase, thus promoting proliferation and invasion and inhibiting apoptosis in GC cells [128]. This finding may provide a new potential therapeutic target for GC and a new explanation for its occurrence. In addition, effective therapies may be achieved through the modulation of the tRFs cited above, which function within the signal transduction pathway.

### 4.6. Colorectal Cancer

CRC is the third most commonly diagnosed cancer worldwide and the second leading cause of cancer death [129]. The mechanisms of tRFs in CRC remain under investigation. Some tRFs can affect signaling pathways, with carcinogenic or anti-carcinogenic effects. For example, the 5′-half 5′tiRNA-His-GTG can induce cell apoptosis under the regulation of the HIF1α/ANG axis. It reduces the expression of large tumor suppressor kinase 2 (LATS2), a core kinase within the Hippo signaling pathway, and subsequently promotes CRC cell multiplication and tumor growth by inhibiting the Hippo pathway [35]. Research has found that tRF/miR-1280 targets the notch ligand JAG2 to activate the Notch signaling pathway, which enhances CRC tumor stem cell activity, promoting the proliferation and metastasis of CRC [130]. tRFs can also affect the progression of CRC by regulating the transcription process. Specifically targeting the SPIB, 5′tiRNA-Gly-GCC regulates the promoter region of signal transducer and activator of transcription 6 (STAT6) and stimulates STAT6 transcription, as well as positively modulating the cJAK1/STAT6 signaling pathway, which collectively contributes to CRC progression [131]. Influenced by tumor-associated mesenchymal cells within the TME, tumor cells need to undergo the EMT to become metastatic. An elevated expression of Claudin accelerates EMT formation [132].

Given the differential expression of tRFs in CRC patients, a growing body of research has been dedicated to investigating their utility as potential biomarkers and developing new diagnostic methods. One study chose tRF-22/27/32 as a plasma marker for CRC diagnosis. In an analysis of the plasma RNA from CRC patients and healthy individuals, these tRFs showed a higher diagnostic value than traditional tumor markers [133]. The combination of tRFs and traditional markers may lead to a more effective diagnostic result. When combined with CEA and CA199, the sensitivity and specificity of 5′-tRFGlyGCC in distinguishing CRC patients were 86% and 84%, respectively, revealing its clinical application potential [134]. The diagnostic efficacy of upregulating tRF-Ala-AGC-060, downregulating tRFTyr-GTA-081, and combining them with CEA for CRC screening was superior compared to individual diagnostic methods [135].

Currently, tRFs can be used for CRC detection and prognosis prediction in patients. The level of i-tRF-GlyGCC in CRC tissues is significantly lower than that in normal tissues, and a higher level of i-tRF-GlyGCC is associated with shorter disease-free survival (DFS) and OS periods, as well as with an increased risk of recurrence [136]. A recent study found that tRFdb-3013a and tRFdb-3013b are significantly decreased in colon and rectum adenocarcinomas; these decreases are significantly associated with a worse clinical survival rate and a shortened OS period in CRC patients, showing their potential as novel biomarkers for CRC prognosis [137]. Another study has pointed out that two kinds of 3′tRF can be employed as diagnostic indicators of CRC, in combination with CEA and CA724 [138].

Radiotherapy and chemotherapy serve as critical strategies in CRC treatment. A substantial downregulation of tRF-16-7 × 9PN5D was found in radioresistant CRC cells, pointing to a link between tRNA and cancer radiation resistance [139]. Also notable is the significant association between the intestinal microbiota, including Escherichia coli, and CRC [140]. 5′-tRF-Leu (CAA), derived from non-pathogenic Escherichia coli, is able to suppress CRC cells [141]. In chemotherapy, quercetin has been found to change the sensitivity of CRC to the conventional chemotherapeutic agent 5-FU through tRFs and to regulate the expression of some specific tRFs [142,143]. However, as we mentioned above, the various tRFs have different functions; some of them could therefore have adverse roles in CRC treatment. Further studies are warranted to confirm which tRFs are critical in this regard.

### 4.7. Leukemia

Studies have also begun to uncover tRF signatures in leukemia. Veronica Balatti et al. demonstrated that the dysregulation of the T-cell leukemia/lymphoma 1 (TCL1) oncogene is a critical contributing event in the pathogenesis of B-cell chronic lymphocytic leukemia (CLL). Furthermore, they found that ts-53 targets the 3′UTR of TCL1, a key oncogene in the development of aggressive CLL and that its downregulation in leukemic cells is inversely correlated with TCL1 expression, contributing to CLL progression [144]. Together with ts-53, ts-101, ts-46, and ts-47 are also downregulated in CLL [74]. Similarly, another report indicated that, compared with normal B cells, both ts-43 and ts-44, derived from distinct transcripts of pre-tRNAHis, are downregulated in CLL; this was also the case for tRF-5, which is derived from tRNAHis [145]. The studies cited above indicate that dysregulations of tRFs are both ubiquitous in CLL and related to tumorigenesis. Furthermore, Katerina Katsaraki et al. discovered tRF-Leu^AAG/TAG^, a particular tRF-3 derived from tRNA^LeuAAG^ and tRNA^LeuTAG^, and revealed that high tRF-Leu^AAG/TAG^ levels are associated with inferior OS in CLL patients. Using stratified Kaplan–Meier OS analysis, they further uncovered an unfavorable prognostic role of high tRF-Leu^AAG/TAG^ levels for patients in Binet A or Rai I stage, as well as with negative CD38 expression and a mutated or unmutated IGHV genomic locus [146].

Apart from CLL, tRFs also play a vital role in other types of leukemia. HTLV-1 is the causative agent of adult T-cell leukemia/lymphoma (ATLL). Reverse transcription is considered a key event in the retroviral life cycle. A recent study determined that a 3′18 nt tRNAPro-derived fragment (tRFPro), packaged into HTLV-1 particles, serves as an RT primer in vitro [147]. These findings indicate that tRFPro could represent a novel target for therapies aimed at controlling HTLV-1 infection [63]. Roy L. Maute et al. discovered that CU1276, a novel tRF, is abundant in normal germinal center B cells but absent in germinal center-derived lymphomas, suggesting that it may play a role in the pathogenesis of this disease. Furthermore, CU1276 suppresses proliferation and modulates the molecular response to DNA damage due to the repression of endogenous RPA1, an essential gene involved in many aspects of DNA dynamics [148]. Researchers also found that pseudouridylation (Ψ) of a stem cell-enriched tRF subtype, specifically of mini tRFs containing a 5′ TOG, selectively inhibits aberrant protein synthesis programs through disturbing the recruitment of translational co-activator PABPC1-interacting protein 1 (PAIP1), thereby strongly repressing translation. The dysregulation of 5′ TOG is clinically associated with leukemic transformation and reduced patient survival. These findings define a critical role for tRFs and Ψ in difficult-to-treat subsets of myelodysplastic syndrome (MDS) characterized by a high risk of progression to acute myeloid leukemia (AML) [149].

### 4.8. Lung Cancer

Lung cancer was the most frequently diagnosed cancer in 2022, responsible for almost 2.5 million new cases, or one in eight cancers worldwide (12.4% of all cancers globally) [129]. However, further research is needed on the role of tRFs in lung cancer, especially their underlying molecular mechanisms. A previous study indicated that ts-53, ts-101, ts-46, and ts-47 are not only downregulated in CLL but also in lung cancer, acting as tumor suppressor factors [144]. Ilias Skeparnias et al. screened two novel tRFs, tRF-3021a (deriving from either tRNAAlaUGC or tRNAAlaCGC) and tRF-5003b (deriving from tRNAGlyGCC), which are, respectively, downregulated and upregulated in lung adenocarcinoma biopsy specimens and are associated with cancer progression [150].

Jiankui Ye et al. uncovered that tRF-16 directly interacts with the insulin-like growth factor 2 mRNA-binding protein 1 (IGF2BP1), decreases its binding to the m6A region on carnitine palmitoyltransferase 1A (CPT1A) mRNA, and downregulates CPT1A transcriptionally. As a result, fatty acid metabolism in lung cancer cells is inhibited, leading to an inhibition of lung cancer cell proliferation in vivo [151]. Another tRF, AS-tDR-007333—a 28 nt long tRF-5 cleaved at sites 1 to 28 on the 5′ end of tRNA-Gly-GCC—was identified in pre- and postoperation plasma from patients with non-small cell lung cancer (NSCLC). AS-tDR-007333 is significantly upregulated in NSCLC tissues, plasma, and cells and is clinically associated with poorer prognosis. A further study demonstrated that AS-tDR-007333 overexpression enhances the proliferation and migration of NSCLC cells by activating mediator complex subunit 29 (MED29) through two different pathways. Firstly, interacting with HSPB1, AS-tDR-007333 enhances H3K4me1 and H3K27ac in the MED29 promoter, thereby activating MED29 expression. Secondly, AS-tDR-007333 increases the transcription of the MED29 promoter by stimulating the expression of transcription factor ELK4 [152].

Recent studies have cast light on the diagnostic and therapeutic potential of tRFs in lung cancer. Fang Hu et al. showcased that tsRNA-5001a is significantly upregulated in lung adenocarcinoma tissues and positively correlated to the risk of postoperative recurrences and poor prognosis in patients [153]. Jipeng Li et al. reported that serum tRF-31-79MP9P9NH57SD expression was higher in NSCLC patients, while the levels of tRF-31-79MP9P9NH57SD were linked to the clinical stage (*p* = 0.002) and lymph node malignancy (*p* = 0.012) [154]. Gu et al. developed a TRY-RNA signature composed of tRFs, rRNA-derived small RNAs, and YRNA-derived small RNAs from human peripheral blood mononuclear cells. The signature precisely discriminates between healthy controls, lung cancer, and pulmonary tuberculosis [155]. Baibing Zheng et al. discovered that the expression levels of exosomal tRF-Leu-TAA-005, tRF-Asn-GTT-010, tRF-Ala-AGC-036, tRF-Lys-CTT-049, and tRF-Trp-CCA-057 were significantly downregulated in NSCLC patients. This significant difference suggests that these five exosomal tRFs may be promising diagnostic biomarkers for NSCLC [156].

### 4.9. Other Cancers

tRFs have also been reported in other cancers, including ovarian cancer, cervical cancer, bladder cancer, and renal cancer.

In ovarian cancer, differentially expressed tRFs were found in high-grade serous ovarian cancer (HGSOC), in which they were involved in protein phosphorylation, transcription, cell migration, the cancer pathway, and the MAPK and Wnt signaling pathways. Importantly, tRF-03357 promoted *SK-OV-3* cell proliferation, migration, and invasion by downregulating the expression of homeobox containing 1 (HMBOX1), a transcription factor related to the development of several tumors, at the transcriptional level [157]. Another study focused on the clinical benefits of tRFs in ovarian cancer, highlighting a significant association of i-tRF-GlyGCC with advanced International Federation of Gynecology and Obstetrics (FIGO) stages, suboptimal debulking, and, most importantly, with early progression and poor OS of epithelial ovarian cancer (EOC) patients [158]. Similarly, 3′U-tRF^ValCAC^, derived from pre-tRNA^ValCAC^, is also significantly associated with poor OS in ovarian cancer. More precisely, a survival analysis of two cohorts, both exceeding a 6-year median follow-up, demonstrated that patients with increased 3′U-tRFValCAC tumor levels are at a significantly higher risk for early progression and poor OS following cytoreductive surgery and first-line platinum-based chemotherapy [159].

In cervical cancer, Yang Wang et al. identified tRF-Glu49 as a potential tumor suppressor gene in cervical carcinoma. tRF-Glu49 inhibited cervical cell proliferation, migration, and invasion processes by directly downregulating the transcription of the oncogene fibrinogen-like protein-1 (FGL1) [150]. On the contrary, two 5′-tRFs, 5′tDR-GlyGCC and 5′tDR-GlnCTG, were found to enhance tumor progression in cervical cancer by promoting ribosome assembly and preventing cell apoptosis triggered by Cyt c [160]. Further research is needed to reveal the underlying mechanism of tRFs in the pathogenesis and progression of cervical cancer.

In bladder cancer, diagnosis is primarily made using invasive tests; however, an increasing number of studies have been devoted to exploring tRFs as screening and diagnostic biomarkers for this disease. For example, one 5′-tRF, 5′-tRF-LysCTT, is associated with an aggressive tumor phenotype, early progression, and poor survival; its integration with clinically available markers could achieve superior specificity and improve the prediction of disease progression for bladder cancer [161]. The circulating tRF-1:28-chrM.Ser-TGA and tiRNA-1:34-Glu-CTC-1-M2 are specifically expressed by bladder cancer cells and are positively associated with the degree of malignancy; they can not only be applied as new biomarkers but are also expected to provide novel targets in the therapy of bladder cancer [162]. tRFs have also demonstrated potential in the treatment of bladder cancer. A novel m7G-modified tRF, m7G-3′-tiRNA LysTTT (mtiRL), has been defined as a critical molecule to promote bladder cancer malignancy in vitro and in vivo. Targeting this tRFan can therefore be an efficient way to treat bladder cancer [163].

To date, studies investigating the role of tRFs in renal carcinoma have been relatively limited, although certain tRFs have been implicated in modulating the tumorigenesis and progression of renal cancer. In clear-cell renal cell carcinoma (ccRCC), one study determined that 5′tRNA4-Val-AAC expression was inversely correlated with the stage and grade of the tumor [164]. Another investigation found that four kinds of 5′-halves were downregulated in serum and tissue in ccRCC. Their lower expression not only indicates a potential role of these 5′-halves as tumor suppressors but also suggests their potential as novel ccRCC biomarkers [165].

## 5. Conclusions and Perspectives

With the application of novel technologies, such as high-throughput transcriptome sequencing, research on tRFs has grown tremendously, while their diversity and functional roles have also been progressively elucidated. Here, we have reviewed the key roles of tRFs, including nascent RNA silencing, translation regulation, epigenetic gene silencing, and reverse transcription inhibition.

Increasing evidence has indicated that tRFs are associated with many aspects of various kinds of cancer, including the generation, progression, and migration of tumors. In this review, we have summarized the roles of tRFs in several cancer types, such as BC, Pca, GC, liver cancer, CRC, PC, and prostate cancer. The underlying mechanisms of how tRFs regulate tumors are still incompletely understood. Meanwhile, the biological functions of these tRFs seem to differ depending on the cancer type in question. Furthermore, the characteristics of these distinct types of cancers, including growth patterns, metastatic behaviors, and tumor microenvironment heterogeneity, drive the production of different tRFs, which exert varied functional roles [22]. For example, hypoxia-induced tRFs play important roles in BC [31]. According to their expression levels, some specific tRFs can be applied as novel non-invasive biomarkers for the diagnosis and detection of cancer and as therapeutic targets for clinical applications.

There are still some limitations in tRF-related research. The nomenclature of tRFs is debated, leading to inconsistencies in publications and confusion among readers. Some tRFs are named by the order of discovery, and this difference may hinder identifying relevant publications [23,74]. To promote the standardization of existing research, as well as in the interest of convenience, a consensus and a common method in tRF nomenclature must be established. Although several tRFs that are dysregulated in cancer have already been characterized, their underlying mechanisms of action require further investigation; this is particularly the case for the determination of causal relationships. Arriving at a successful utilization of tRFs in the clinic as biomarkers for cancer will be a long process. Their potential for cancer diagnosis has been emphasized; however, the detection of tRFs mainly relies on high-throughput sequencing and other innovative methods, which are relatively expensive or too complicated for large-scale clinical detection. Their stability and accuracy may also need to be improved, given the interaction of tRFs with other substances. Therefore, more attempts to develop appropriate tRF testing methods are urgently needed to improve their efficacy in clinical detection.

As a novel type of ncRNA, tRFs have shown potential in the diagnosis and treatment of cancer. However, significant limitations persist. Further investigations remain imperative to reveal the mechanisms of tRFs in cancers, confirm their most crucial elements, and utilize them as clinically actionable biomarkers and innovative therapeutic targets.

## Supplementary Materials

The following supporting information can be downloaded at: https://www.mdpi.com/article/10.3390/ijms26125822/s1.
