# Supplementary material for: The Roles of tRNA-Derived Fragments in Cancer: Updates and Perspectives
Source: Int J Mol Sci. 2025 Jun 17;26(12):5822. doi: 10.3390/ijms26125822 (PMC12192838; doi:10.3390/ijms26125822)
Supplement: Supplementary file 1 [file ijms-26-05822-s001.zip › ijms-3621420-supplementary.pdf]

Table S1. Functional tRFs in different types of cancer

Cancer type	tRF name	Role	Function	Ref
Breast cancer (BC)	5'-tRF-His-GTG	downregulate in BC plasma samples	potential biomarker of BC	[72]
	tRF-ArgCCT-017, tRF-Gly-CCC-001, tiRNA-Phe-GAA-003	upregulate in BC plasma samples	potential biomarker of BC	[73]
	tRF-19-W4PU732S	oncogenic factor	promote proliferation and malignance of BC and suppress apoptosis by inhibiting RPL27A	[75]
	5′-SHOT-RNA	oncogenic factor	promote proliferation of BC	[76]
	ts-112	oncogenic factor	promote proliferation of BC	[77]
	tRF-33	oncogenic factor	disrupt mitochondrial homeostasis and promote progression of BC by interacting with IGF1	[78]
	5′-tRF-GlyGCC	oncogenic factor	promote tumorigenesis of BC at epigenetic level	[79]
	tRFGlu, tRFAsp, tRFGly, tRFTyr	tumor suppressor	inhibit metastasis of BC via YBX1 displacement	[80,81]
	5′-tiRNAVal	tumor suppressor	inhibit the FZD3-mediated Wnt/β-Catenin signaling pathway in BC	[82]
	tRF3E	tumor suppressor	inhibit proliferation of BC through NCL-mediated mechanism	[83]
	tDR-000620	downregulate in TNBC CSCs and serum sample	potential biomarker of TNBC	[84]
	tRFLys-CTT-010	oncogenic factor	promote proliferation of TNBC by regulating glucose metabolism via tRFLys-CTT-010/G6PC axis	[85]
	tDR-0009, tDR-7336	drug-resistance driver	facilitate doxorubicin resistance in TNBC	[86]
	tRF-27	oncogenic factor drug-resistance driver	facilitate trastuzumab resistance and promote cell proliferation in BC	[87]
	3′tRF-AlaAGC	drug-resistance driver	enhance the Adriamycin sensitivity in BC via NF-κb signaling pathway by silencing 3′tRF-AlaAGC	[89]
Prostate cancer (PCa)	tRF-1001	oncogenic factor	promote proliferation of PCa	[23]
	5′-SHOT-RNA	oncogenic factor	promote proliferation of PCa	[76]
	tRF-315	drug-resistance driver	facilitate cisplatin resistance in PCa	[96]
	5′TOGs	tumor suppressor	inhibit proliferation of PCa	[97]

Table S1. Cont.

Cancer type	tRF name	Role	Function	Ref
Pancreatic cancer (PC)	tRF-Leu-AAG	oncogenic factor	promote proliferation, migration, and invasion of PC by downregulating UPF1	[98]
	tRF-19-PNR8YPJZ	oncogenic factor	promote migration and invasion of PC via AXIN2 axis	[99]
	tRF-18-8R6546D2	oncogenic factor	promote malignancy of PC by silencing ASCL2 and regulating MYC and CASP3	[100]
	tRF-19-Q1Q89PJZ	tumor suppressor	inhibit proliferation and metastasis of PC by suppressing HK1 expression	[101]
	tRF-GluCTC-0005	oncogenic factor	promote proliferation, migration and invasion of PDAC and liver metastasis	[103]
	tRF-21-VBY9PYKHD	tumor suppressor	promote proliferation, migration, and invasion of PDCA when downregulated	[70]
	tRF-Pro-CGG	downregulate in advanced PC	potential biomarker of PC	[104]
Liver cancer	LeuCAG3′tsRNA	oncogenic factor	increase viability of HCT-116 cell by interacting with RPS28 and RPS15	[51]
	Gly-tRF	oncogenic factor	increase LCSC subpopulation proportion and promote EMT in liver cancer	[105]
	HCETSR	tumor suppressor	inhibits HCC malignancy by regulating the SPBTN1-catenin complex axis	[107]
	tRNA-ValTAC-3, tRNA-GlyTCC-5, tRNA-ValAAC-5, tRNA-GluCTC-5	upregulate in liver cancer	potential biomarker of liver cancer	[109]
	tRF5-GlyGCC	radiotherapy inhibitor	reduce NK cell cytotoxicity and limit radiotherapeutic efficacy	[115]
Gastric cancer (GC)	hsa_tsr016141, tRF31-U5YKFN8DYDZDD, tRF-23-Q99P9P9NDD	oncogenic factor	upregulation is associated with tumor grade, lymph node metastasis and invasion	[116–118]
	tRF-3017A	oncogenic factor	promote migration and invasion of GC by targeting NELL2	[9]
	tRF-3019a	oncogenic factor	promote proliferation, migration and invasion of GC	[119]
	tRF-23-Q99P9P9NDD	oncogenic factor	promote progression of GC by affecting lipid metabolism and ferroptosis via targeting ACADSB	[120]
	tRF-33-P4R8YP9LON4VDP, tRF-193L7L73JD	tumor suppressor	inhibit proliferation of GC by disrupting cell cycle	[121,122]
	tRF-5026a	tumor suppressor	inhibit progression of GC via PI3K/AKT signaling pathway and regulate cell cycle	[123]

Table S1. Cont.

Cancer type	tRF name	Role	Function	Ref
	tRF-Glu-TTC-027, tRF-Val-CAC-016	tumor suppressor	inhibit malignance of GC via MAPK pathway	[124,125]
	tRF-Tyr	tumor suppressor	inhibit progression of GC via c-Myc/Bcl2/Bax pathway	[126]
	tRF-Val	oncogenic factor	promote proliferation and invasion and inhibit cell apoptosis of GC	[128]
Colorectal cancer (CRC)	5′tiRNA-His-GTG	oncogenic factor	promote proliferation and inhibit cell apoptosis of CRC	[35]
	tRF/miR-1280	oncogenic factor	promoting proliferation and metastasis of CRC via Notch signal pathway	[130]
	5′tiRNA-Gly-GCC	drug-resistance driver	facilitate 5-FU resistance in CRC	[131]
	tRF-22, tRF-27, tRF-32	upregulate in CRC tissue and plasma samples	potential biomarker of CRC	[133]
	5′-tRFGlyGCC	upregulate in CRC plasma samples	potential biomarker of CRC	[134]
	tRF-Ala-AGC-060	upregulate in CRC tissue samples	potential biomarker of CRC	[135]
	tRFTyr-GTA-081	downregulate in CRC tissue samples	potential biomarker of CRC	[135]
	i-tRF-GlyGCC	downregulate in CRC tissue samples	potential biomarker of CRC	[136]
	tRFdb-3013a, tRFdb-3013b	downregulate in colon and rectum adenocarcinomas	potential biomarker of CRC	[137]
	tRF-16-7X9PN5D	radiosensitizer	promote proliferation, migration, invasion and radio resistance when downregulated	[139]
	5′-tRF-Leu (CAA)	tumor suppressor	inhibit progression of CRC	[141]
Leukemia	ts-53, ts-101, ts-46, and ts-47, ts-43, ts-44	tumor suppressor	promote progression of CLL	[74,144,145]
	tRF-Leu$^{AAG/TAG}$	upregulation is associated with inferior OS	potential biomarker of CLL	[146]
	tRFPro	RT primer of HTLV-1	potential target for preventing ATLL	[147]
	CU1276	tumor suppressor	inhibit proliferation and molecularly modulate DNA damage repair	[148]
	mTOG	dysregulation associated with leukemic transformation	potential biomarker for MDS transformation to AML	[149]

Table S1. Cont.

Cancer type	tRF name	Role	Function	Ref
Lung cancer	ts-46, ts-47	tumor suppressor	inhibit proliferation of lung cancer cell lines when upregulated	[144]
	tRF-3021a	downregulation is associated with cancer progression	potential biomarker for poor prognosis	[150]
	tRF-5003b	upregulation is associated with cancer progression	potential biomarker for poor prognosis	[150]
	tRF-16	tumor suppressor	inhibit fatty acid metabolism and proliferation of lung cancer	[151]
	AS-tDR-007333	oncogenic factor upregulated in NSCLC tissues and plasma	promote proliferation and migration of NSCLC potential biomarker of NSCLC	[152]
	tsRNA-5001a	upregulated in lung adenocarcinoma tissues	potential biomarker for poor prognosis	[153]
	tRF-31-79MP9P9NH57SD	upregulated in NSCLC	potential biomarker for poor prognosis	[154]
	tRF-Leu-TAA-005, tRF-Asn-GTT-010, tRF-Ala-AGC-036, tRF-Lys-CTT-049, and tRF-Trp-CCA-057	downregulated in NSCLC tissues	potential biomarker of NSCLC	[156]
ovarian cancer	tRF-03357, 3′U-tRFValCAC	oncogenic factor	promote proliferation, migration and invasion of SK-OV-3 cell	[157,159]
	i-tRF-GlyGCC	upregulation is associated with advanced FIGO stages	potential biomarker of ovarian cancer	[158]
cervical cancer	tRF-Glu49	tumor suppressor	inhibit proliferation of cervical carcinoma by regulating FGL1	[150]
	5′tDR-GlyGCC, 5′tDR-GlnCTG	oncogenic factor	promote progression of cervical cancer	[160]
bladder cancer	5′-tRF-LysCTT	upregulation is associated with poor prognosis	potential biomarker of bladder cancer	[161]
	tRF-1:28-chrM.Ser-TGA, tiRNA-1:34-Glu-CTC-1-M2	oncogenic factor	promote malignancy of bladder cancer	[162]
	m7G-3'-tiRNA LysTTT (mtiRL)	oncogenic factor	promote malignancy of bladder cancer	[163]
renal carcinoma	5′tRNA4-Val-AAC	downregulation is associated with advanced stage	potential biomarker of ccRCC	[164]
	5'-tRNA-Arg-CCT, 5'-tRNA-Glu-CTC, 5'-tRNA-Leu-CAG and 5'-tRNA-Lys-TTT	downregulated in tumor tissues and serum samples	potential biomarker of ccRCC	[165]
